# Addressing data and methodological limitations in estimating catastrophic health spending and impoverishment in India, 2004–18

**DOI:** 10.1186/s12939-021-01421-6

**Published:** 2021-03-20

**Authors:** Sanjay K. Mohanty, Laxmi Kant Dwivedi

**Affiliations:** 1 Department of Fertility Studies, International Institute for Population Sciences, Mumbai, India; 2 Department of Mathematical Demography and Statistics, International Institute for Population Sciences, Mumbai, India

**Keywords:** Catastrophic health spending, Impoverishment, India, Capacity-to-pay

## Abstract

**Background:**

Estimates of catastrophic health expenditure (CHE) are counterintuitive to researchers, policy makers, and developmental partners due to data and methodological limitation. While inferences drawn from use of capacity-to-pay (CTP) and budget share (BS) approaches are inconsistent, the non-availability of data on food expenditure in the health survey in India is an added limitation.

**Methods:**

Using data from the health and consumption surveys of National Sample Surveys over 14 years, we have overcome these limitations and estimated the incidence and intensity of CHE and impoverishment using the CTP approach.

**Results:**

The incidence of CHE for health services in India was 12.5% in 2004, 13.4% in 2014 and 9.1% by 2018. Among those households incurring CHE, they spent 1.25 times of their capacity to pay in 2004 (intensity of CHE), 1.71 times in 2014 and 1.31 times by 2018. The impoverishment due to health spending was 4.8% in 2004, 5.1% in 2014 and 3.3% in 2018. The state variations in incidence and intensity of CHE and incidence of impoverishment is large. The concentration index (CI) of CHE was − 0.16 in 2004, − 0.18 in 2014 and − 0.22 in 2018 suggesting increasing inequality over time. The concentration curves based on CTP approach suggests that the CHE was concentrated among poor. The odds of incurring CHE were lowest among the richest households [OR 0.22; 95% CI: 0.21, 0.24], households with elderly members [OR 1.20; 95% CI:1.12, 1.18] and households using both inpatient and outpatient services [OR 2.80, 95% CI 2.66, 2.95]. Access to health insurance reduced the chance of CHE and impoverishment among the richest households. The pattern of impoverishment was similar to that of CHE.

**Conclusion:**

In the last 14 years, the CHE and impoverishment in India has declined while inequality in CHE has increased.

**Supplementary Information:**

The online version contains supplementary material available at 10.1186/s12939-021-01421-6.

## Background

Reliable estimates of catastrophic health expenditure and medical impoverishment are increasingly sought by national and state governments, public health professionals, international organisations, and developmental partners. Catastrophic health expenditure (CHE) is one of the monitoring indicators of health-related SDGs and financial risk protection [1]. Available estimates of CHE are incomplete owing to data and methodological constraints and the use of varying analytical approaches. Varying estimates from the same data source or from different but contemporaneous surveys are counterintuitive to policy makers, planners, and researchers [2–4]. In this paper, we demonstrate how to overcome these constraints by estimating CHE and impoverishment using a unique approach and appropriate data source in the Indian context.

In health economics literature, two methods, namely, the budget share (BS) and capacity-to-pay (CTP) methods are used to estimate catastrophic health expenditure. The BS method defined health expenditure as catastrophic if the household’s out-of-pocket (OOP) payment to household consumption expenditure/income exceeds a pre-defined (threshold) limit [5]. Varying thresholds are feasible and a threshold of 10% is commonly used in defining CHE. The CTP approach defines health expenditure as catastrophic if OOP payments exceeds 40% of capacity to pay [6]. Capacity to pay (CTP) is derived in multiple ways; a) total household expenditure less subsistence expenditure b) total household expenditure less of food expenditure and c) total household expenditure less of poverty line. Each method has some merits and limitations and empirical results from both these approaches in various country settings have been discussed elsewhere [3, 4, 7]. Estimates based on capacity to pay (CTP) are pro-poor, take into account equity concerns, and are recommended by the WHO. This is because loss of welfare due to OOP payments for health care is higher among the poor than among the rich, as poor households resort to borrowing and selling assets, underutilise or do not seek health care due to lack of resources while rich people meet OOP payment through income or savings. However, this method does not capture the absolute hardship associated with OOP payment, does not consider savings/borrowings and life-time consequences of health shock [8]. The budget share approach is easy to compute, required limited data but not sensitive to poor. Literature also suggests that households without health insurance, poor households, elderly households, large households, households in rural areas and households with chronically sick members are more likely to incur CHE [7, 9].

In India, the health surveys (HSs) of 2004 and 2014 and the consumption survey (CSs) of 2004 and 2011–12 – conducted by the National Sample Survey (NSS) – have been the main basis of estimating CHE and provide evidences for national and state policy [10–13]. While the HSs collected comprehensive information on hospitalisation, outpatient visits, health expenditure, and reimbursement, it had canvassed few questions on consumption expenditure. The HSs did not collect data on the food expenditure of households which used to be the basis for estimating CHE in India. The CSs collected detailed information on household consumption. Unlike in the HSs, the questions on health expenditure in the CSs were limited and were canvassed at the household level. Surveys with fewer questions yield lower estimates compared to those with detailed questions [14]. With regard to the suitability of data, the HSs are ideal for estimating CHE, but researchers often cite the non-availability of information on food expenditure as the reason for not using the CTP approach.

A large body of literature suggests that the high OOP payment are positively associated with CHE and distress financing, and increases the probability of poverty and impoverishment [15–21]. The risk of CHE is said to be higher among the rich [22], possibly due to choice of methods. Impoverishment, defined an increase in poverty level owing to OOP payment for health care is an important measure of financial protection. The impoverishment effect of health spending are estimated using international, national and regional poverty lines or from subsistence expenditure along with household income/consumption expenditure [19, 23]. About 12 million households in India (accounting for 6.2% of the population in 2004) were impoverished due to health spending and impoverishment was higher for those using outpatient care than inpatient care [24]. Over two-thirds of household resort to OOP payment for health care in India and it remained high over time [25, 26]. A growing number of studies have highlighted the high OOP payment for varying health services in India [27, 28]. Among others, low insurance coverage, high cost of treatment, increased income level, low public spending on health, type of diseases and increased use of private health centres are leading factors of high OOP [29, 30].

In response to the high OOP payments, CHE and low health care utilisation, the Government of India and the states governments have implemented several schemes in recent decades. Among others, the National Health Mission (NHM) was implemented in 2005; this is the largest conditional cash transfer programme worldwide and has been successful in improving maternal care and reducing CHE for maternal care throughout the country [31]. The *Ayushman Bharat Yojana (ABY),* implemented in 2018, is the largest-ever publicly funded financial protection scheme world (www.ayushmanbharat.co.in/). The National Health Policy (NHP, 2017) stipulated that central government spending on health should increase to 2.5% of GDP by 2025 and the state government spending to 8% of respective state budget by 2020 and to reduce CHE by 25% by 2025 [32].

Using the NSS data, estimates of CHE in India are available for hospitalisation, outpatient services, health services (inpatient or outpatient), maternal care and specific diseases. Estimates of CHE for health services varied from 4 to 15% in 2004 and from 15 to 25% between 2011 and 14 [2, 27, 33, 34]. Two papers in the WHO bulletin have highlighted large variations in CHE due to the use of varying methods and data sources and suggested standardising the estimates [2, 30]. A number of studies have provided estimates of CHE specific to maternal care; showing large variations [31, 35–37]. Some have provided estimates specific to hospitalisation by disease [29, 38]. Most of these studies on CHE in India used the BS approach, and cited non-availability of data on food expenditure as reasons for not using the CTP approach. Estimates of CHE derived from BS and CTP methods using the same data source are inconsistent [2, 35, 39] and estimates derived from budget share method are also counterintuitive (classify rich people incurring high CHE) [4, 35].

In this paper, we overcome these limitations by linking household consumption and health survey to address the data gap and provided estimates for the incidence and intensity of CHE and impoverishment from the health surveys of 2004, 2014 and 2018 using the capacity-to-pay approach. The contribution of the paper is two-fold. First, it demonstrates how to derive estimates of CHE in the absence of data on food expenditure from the health surveys. Second, it provides comprehensive estimates of CHE based on hospitalisation and outpatient visits over three periods of time in the Indian context. The estimates derived are robust, captured the equity concern and can be replicated elsewhere in a similar situation.

## Methods

### Data

The National Sample Survey is the only data source that provides the opportunity to estimate OOP, CHE and impoverishment periodically in India. We have used unit data from the five cross-sectional nationwide surveys on consumption and health care carried out by the National Sample Survey (NSS), in the country between 2004 and 2018. The consumption surveys (schedule 1.0) of 2004 and 2011–12 (henceforth referred to as 61(1.0) and 68(1.0) respectively) were population-based surveys specifically designed to estimate poverty, inequality, food consumption patterns, calorie intake, and per capita consumption expenditure etc. across states by rural and urban areas. The consumption surveys (CSs) recorded consumption of over 200 food and non-food items and limited information about outpatient and inpatient expenditure, over a reference period of 30 days and 365 days respectively. Data on health expenditure in these surveys was collected at the household level rather than the individual level and information about reimbursement was not collected in these surveys.

The HSs (schedule 25.0) of 2004, 2014 and 2017–18 henceforth referred to as 60(25.0), 71(25.0) and 75(25.0) respectively were population-based health surveys carried out across all states and union-territories in India. The reference period for hospitalisation (365 days) and outpatient visit (15 days) was uniform in all three rounds of the survey. Typically, the HSs record expenditure on health for each episode of hospitalisation, expenditure on maternal care (antenatal, natal, post-natal and immunisation) and reimbursement for member of households and provide data at different levels. The comprehensive measures of OOP, CHE and impoverishment typically includes all expenditure on hospitalisation, outpatient visit and maternal care at the household level. Data on household consumption expenditure was collected through a few questions over a 30-day reference period. In general, the estimates derived from CSs and HSs are reliable and conducted at regular intervals of time and the details of the survey findings are available in national reports [10–13]. We have used these five cross sectional surveys due to the close proximity of the survey periods or because they are the most recent survey periods. The NSS surveys used a two-stage stratified sampling approach, with the first sampling units being village and urban blocks and the second being households. All the results including regression analysis were reported after assigning the appropriate weight. The cluster and stratification adjustment was made in Stata using the “svyset” command.

### Outcome variables

Catastrophic health expenditure (CHE) and impoverishment are two outcome variables in the analyses, derived from OOP payment for hospitalisation and outpatient visits. The analytical strategy was to capture the aggregate health expenditure of households as some households had spent on hospitalisation only, some on outpatient care only, some on both and some did not use health services. The NSS data is usually analysed for hospitalisation or outpatient visits and not aggregated at household level; our estimates are at household level. We have aggregated healthcare payments at the episode level (separately for hospitalisation and outpatient visits), derived estimates at the individual and household level to derive household expenditure and reimbursement. OOP payments for inpatient and outpatient care were standardised to 30 days period as household consumption expenditure data was available on a monthly basis. OOP payments for health services were derived by combining payment for both hospitalisation and outpatient services including maternal care. The antenatal, natal, post-natal and immunisation received are included in inpatient care as these services are received almost a year. Our variable for health expenditure includes expenditure on medicine, diagnosis tests, bed charges, physician’s fees, transportation and other expenses.

### Statistical methods

A number of methods were used for estimating incidence and intensity of CHE, impoverishment, estimation of concentration index and concentration curve and determinants of CHE. A brief description of each of methods used is provided below.

### Incidence and intensity of CHE using the capacity-to-pay approach

The incidence and intensity of CHE were computed in two steps. In the first step, a household was classified as incurring CHE based on their consumption expenditure (C), OOP payments for health care (OOP), and subsistence expenditure (SE). In micro data, a household was dichotomised as incurring catastrophic health expenditure (1 = yes and 0 = no) if their OOP exceeded 40% of capacity to pay [40]. In the second step, the households were aggregated in order to derive the incidence of CHE. The threshold of CHE of a household is defined as

1 $$ {\mathrm{E}}_{\mathrm{i}}=\mathrm{OOPi}/{\left({\mathrm{C}}_{\mathrm{i}}-{\mathrm{SE}}_{\mathrm{i}}\right)}_{=}{\mathrm{OOP}}_{\mathrm{i}}/{\mathrm{ctp}}_{\mathrm{i}}>0.4 $$

Where ctp is capacity to pay of household. Ei is binary; takes value of 1 if household incurred CHE and 0 otherwise.

Subsistence expenditure (the minimum expenditure of the household for meeting basic needs) was computed as the median food expenditure adjusted by the equivalent household size.

Incidence is estimated based on all sample households (N).

2 $$ \mathrm{Incidenceof}\mathrm{CHE}=1/\mathrm{N}\sum \limits_{.}^{.}\mathrm{Ei} $$

Intensity is computed based on those households who had incurred the CHE and defined as

3 $$ \mathrm{Intensityof}{\mathrm{CHE}}_{\mathrm{i}}=1/\mathrm{U}\sum \limits_{.}^{.}\left(\left({\mathrm{OOP}}_{\mathrm{i}/}{\mathrm{CTP}}_{\mathrm{i}}\right)-0.4\right)\Big) $$

Where U is the number of households incurring CHE. The intensity of CHE is estimated among those households incurring CHE [41–43]. The intensity of CHE is estimated only for India and not by states due to lower cell size.

### Estimation of impoverishment

A non-poor household was said to be impoverished by health payments if their consumption expenditure fell short of subsistence expenditure following health expenditure [40], i.e.

4 $$ {\mathrm{IMPO}}_{\mathrm{i}}:{\mathrm{C}}_{\mathrm{i}}>{\mathrm{SE}}_{\mathrm{i}}\mathrm{and}\ {\mathrm{C}}_{\mathrm{i}}-{\mathrm{OOP}}_{\mathrm{i}}<{\mathrm{SE}}_{\mathrm{i}-} $$

where IMPO is impoverishment, C is household consumption expenditure before OOP payment and SE is subsistence expenditure.

The incidence of impoverishment was estimated by summing up the impoverished households over all sample households.

#### Incidence and intensity of CHE using the budget share approach

In computing CHE using the budget share (BS) approach, a household was classified as incurring CHE if their OOP for health care exceeded 10% of household consumption expenditure, i.e.

5 $$ {\mathrm{CHE}}_{\mathrm{i},\mathrm{BS}}=\mathrm{OOPi}/\mathrm{Ci}>0.1 $$

Where the sub-script i refers to the ith household.

The incidence and intensity was computed in a similar way as defined in Eq. (2) and (3).

### Estimates at constant prices

All price sensitive estimates (MPCE, OOP payments, and reimbursements) were presented at 2018 prices in order to facilitate comparison over time. The state-specific price index (agricultural labourer for rural areas and industrial workers for urban areas) were used in deriving the comparable estimates over time.

### Procedure of estimating CHE and Impoverishment from NSS data

CHE and impoverishment were estimated using the CTP approach. To estimate CHE, we assumed that the share of per capita food expenditure to per capita consumption expenditure by states and place of residence has remained similar in recent times. It should be mentioned that the share of per capita food expenditure to consumption expenditure is a key and stable economic indicator. In the short run this indicator is less likely to change though it will decline in long run (say five years or more) with the expected increase in income level of the population. The consumption survey was of 2004 and health survey of 2004 were in same year. The consumption survey in 2011–12 and health surveys were in 2014 were with a difference of 2 year. However, there was a difference of about 6 years between 2011 and 12 and 2017–18 health survey. Since no latest consumption survey was available between we kept the distribution constant and mentioned in the limitations. Besides in derivation of CHE, we have used the subsistence expenditure. For those households for whom the household consumption expenditure is lower than the subsistence expenditure, any OOP for health services is catastrophic. Based on these assumptions, we used the following steps to derive subsistence expenditure and the capacity to pay from the health surveys.

- **Step 1:** Estimate share of per capita food expenditure (median) to monthly per capita consumption expenditure (median per capita consumption expenditure) from the nearest consumption survey year, i.e. 2004 and 2011–12, by each states and for rural and urban areas.
- **Step 2:** Regress household food expenditure and household size for urban and rural areas separately (from consumption surveys) for 2004 and 2011–12.
  ln _ food_i_ = a + b ∗ ln  _ hsize_i_ +  ∑ c_j_state_i_ + e (6)

where ln_food_i_ is the logarithmic transformation of per capita food expenditure of i^th^ household, ln_hsize_i_ is the logarithmic transformation of i^th^ household size, and state_i_ is the i^th^ state/ union territory.

- **Step 3:** Impute the share of food expenditure from consumption survey to state-specific per capita consumption expenditure from the health survey, separately for rural and urban areas, in order to obtain food expenditure in the health surveys
- **Step 4:** Adjust the food expenditure to the equivalent household size in health survey data to obtain household subsistence expenditure
- **Step 5:** If household consumption expenditure is lower than subsistence expenditure, treat the household health expenditure as catastrophic.
- **Step 6:** The incidence of CHE is estimated by using all sample households in the denominator irrespective of use of health services.

### Estimation of concentration index and derivation of concentration curves

Concentration indices are increasingly used to measure health inequality in population The concentration index varies between − 1 and 1; the closer the value is to − 1, CHE is concentrated among the poor (pro-poor); the closer the value is to + 1, the CHE is concentrated among the rich (pro-rich). The detail methods of estimation of concentration index can be found elsewhere [44, 45]. The concentration index of CHE using both the methods was estimated and compared.

### Logistic regression analyses

The logistic regression analysis was used to understand the significant predictors of CHE on health care and impoverishment in India. Our dependent variable was the use of any health services by the household. Data from health surveys of 2004, 2014 and 2018 were polled in order to understand the effect of time on CHE and impoverishment. The analysis of pooled cross section data account for secular differences in the variables over time. In fact, in addition to increasing the sample size, the point of a pooled cross-sectional analysis is to ascertain how a key relationship has changed over time [46].

The set of covariates used were MPCE quintile, place of residence, household size, health insurance coverage, type of main employment of household, demographic characteristics of head of household, presence of elderly member(s) in the household, type of health services (inpatient care, outpatient care and both inpatient and outpatient care) religion of head of households. The inclusion of these variables in the analyses was based on the literature and the availability of data in various health surveys. MPCE is a direct measure of the economic well-being of households and was used in the absence of income data. We categorised MPCE into five quintiles; poorest, poorer, middle, richer and richest. Place of residence was dichotomised as rural or urban. Household size was reclassified into three categories; 1–4, 5–7 and 8+. The type of main employment of the household was recategorized as labourer, self-employed, wage/salary and others. Of these, the wage and salary are the better off groups while labourers are the poorer group in the population. The age of the head of the household was measured in years and re-categorised as under 30, 30–44, 45–59 and 60+. Similarly, the sex of the household head was classified as either male or female, as reported in the survey. The education level of the household head was categorised as no education (none), up to primary (5 years of schooling), up to secondary (6–10 years) and higher secondary and above (10+ years). Furthermore, we computed a variable to identify if any member in the household was over 60 years (elderly) and included in the analyses. The religion of the household head was categorised as Hindu, Muslim, Christian, Sikh and other. Health insurance is one of the key variables in reducing CHE and impoverishment but the health insurance variable was not uniform over all three health survey rounds. For this reason, we reclassified health insurance to dichotomous variables; households covered by any health insurance and households not covered by health insurance. We created the interaction term of time and all the selected covariates in order to capture the changing nature of the covariates over time if any. The interaction term of time, insurance coverage and MPCE quintile were also used in the model to help understand whether insurance helped to reduce CHE across the economic quintile over time. Adding interaction between time and all selected covariates in the multivariate logistic regression analysis is equivalent to the multilevel random slope model where observations are nested within different time periods [47].

We have presented our results in five sections; section 1 presents descriptive statistics. Section 2 presents estimation of capacity to pay, section 3 presents estimates of CHE and impoverishment, section 4 presents concentration curves and indices and section 5 presents determinant of CHE and impoverishment.

## Results

### Descriptive statistic

Table 1 presents set of key variables from consumption and health surveys between 2004 and 2018 at 2018 prices. The overall use of health services (either inpatient or outpatient) has increased between 2004 and 14 and remained similar in 2018. About three-fifth households used inpatient care including maternal care in 2018. The estimates of inpatient care from consumption survey was lower than that of health survey. Similarly, about one-third of households availed the out-patient care in 15 days reference period (health survey) and it was higher in consumption surveys. Reference period of outpatient care was 15 days for health survey and 30 days for consumption survey. The household size from both the surveys were similar and has been declining over time. During 2004–18, the MPCE (both mean and median) at constant prices has increased by about 33% (from HS) and similar increase was also found during 2004–12. Food expenditure– the basis of computing subsistence expenditure – was about 62% of per capita consumption expenditure in 2004 and declined to 55% by 2011–12. This is expected as the share of food expenditure is likely to decline with improvement in living standard of the population. The proportion of households who had availed hospitalisation or outpatient visit has increased during 2004 and 2014 and remains similar in 2018. The mean OOP payment on hospitalisation of the household (from health surveys) has increased by 60% during 2004–14 while it has declined by 20.5% during 2014–18 (at 2018 prices). Among those availed reimbursement, the mean reimbursement has increased by 56% during 2004–18; 19.6% between 2004 and 14 and 30.5% between 2014 and 18.

**Table 1:** Descriptive statistics from various rounds of health and consumption survey in India, 2004–18

Variables/ Characteristics	Health Survey			Consumption Survey	
	2004	2014	2018	2004	2011–12
Number of households covered	73,868	65,932	113,823	124,644	101,651
Percentage of household availed inpatient care	43.09	72.68	73.23	10.72	15.87
Percentage of household availed outpatient care	36.51	38.35	27.50	61.39	75.85
Percentage of household availed either inpatient or outpatient care	59.36	81.35	79.94	64.81	79.14
Mean Household Size	4.82	4.51	4.35	4.74	4.42
Monthly per capita Consumption Expenditure (MPCE) in Indian Rupees at 2018 prices (95% CI)	1516	1915	2162	1728 (1716–1740)	2542 (2526–2559)
Food expenditure as share of monthly per capita consumption expenditure (median)	NA	NA	NA	61.81	54.72
Mean expenditure on hospitalization (in Indian rupees) of households in 365 days* at 2018 prices (95% CI)	15,311	24,561	19,574	17,996 (17270–18,722)	19,386 (18657–20,115)
Mean reimbursement of household on hospitalization in 365 days at 2018 prices (in Indian Rupees)	1116	1335	1742	NA	NA

NA: Not available

* Among those incurred any expenditure and does not exclude reimbursement

### Estimation of household subsistence expenditure

One of the key contribution of this exercise was the derivation of the subsistence expenditure of households in India. Table 2 presents the coefficient estimated by regressing food expenditure and household size for 2004 and 2011–12, derived from unit data. The coefficient was lower in rural than in urban areas, suggesting that average increase in food consumption for an additional member of the household was lower in the latter than in the former. The coefficient was smaller in 2011–12 than in 2004, for both rural and urban areas.

**Table 2:** Result of regression analyses with monthly per capita consumption expenditure (MPCE) as dependent and household size as independent variable, 2004 & 2011–12

Parameters/N	2004		2014	
	Rural	Urban	Rural	Urban
Coefficient	0.79	0.65	0.72	0.56
Standard error	0.003	0.004	0.003	0.004
Confidence Interval	0.782–0.792	0.647–0.661	0.719–0.730	0.557–0.571
R^2^	0.56	0.44	0.51	0.41
N	79,265	45,303	59,678	41,960

Household subsistence expenditure is the key variable for estimating CHE and impoverishment using the CTP approach. In the CTP approach, subsistence expenditure is defined as the median food expenditure and we used similar concept in our estimation. Median food expenditure as a share of household consumption expenditure, derived from the consumption surveys has declined over time in both rural and urban areas. In rural India, the monthly per capita consumption expenditure (median) was 456 rupees in 2004 and 1198 rupees in 2011–12. Food expenditure accounted for 64% of consumption expenditure in 2004 and 61% in 2011–12. In urban India, the MPCE was 1198 rupees in 2004 and 2019 rupees in 2011–12 and food expenditure accounted for 52% of MPCE in 2004 and 35% in 2011–12. We imputed the median food expenditure from the consumption survey to the health survey. Using these distributions and the median MPCE from the health surveys, the state estimates of median food expenditure were imputed to the health survey data and adjusted by equivalent household size in order to derive the household subsistence expenditure. Subsistence expenditure as a share of consumption expenditure declined in rural India from 44% in 2004 to 40% in both 2014 and 2018. Subsistence expenditure in urban India was 28% in 2004, 18% in 2014, and 19% in 2018 **(**Additional file 1**)**.

**Fig. 1:**
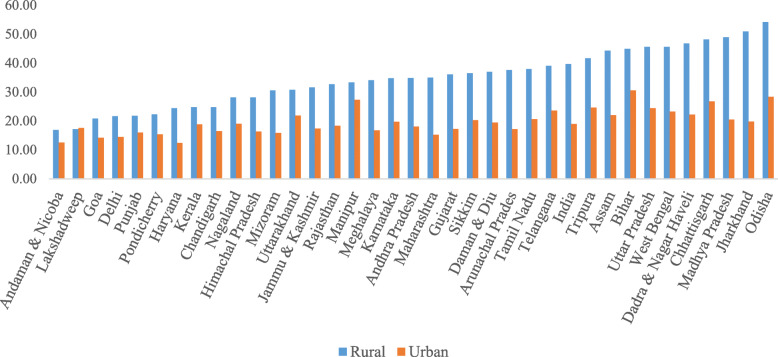
Subsistence expenditure as share of household consumption expenditure in states of India, 2018

Figure 1 presents the state variations in household subsistence expenditure which shows the general pattern of development observed in the states of India. It was lower in rural areas than in urban areas across all states. For example, in 2018, subsistence expenditure in rural Odisha accounted half of household consumption expenditure compared to about one-quarter in urban areas. Similarly, subsistence expenditure as a share of household consumption expenditure was higher in the poorer states of Odisha, Bihar, Chhattisgarh, and Uttar Pradesh and lower in the richer states of Delhi, Goa, Chandigarh, and Kerala in all three period (Additional file 1).

### Variations in OOP, CHE and impoverishment by socio-economic and demographic characteristics in India

Table 3 presents the OOP payment, incidence and intensity of catastrophic health expenditure and impoverishment and the correlation coefficient of CHE for hospitalisation, outpatient care, and health care (either hospitalisation or outpatient visit) for India using two methods at three point of time. The OOP payment by households for hospitalisation and outpatient visits has increased during 2004 and 2014 and declined during 2014 and 2018. The incidence and intensity of CHE and impoverishment due to hospitalisation and outpatient care in India remained similar between 2004 and 2014 and declined between 2014 and 2018. For any health services, the estimates of CHE derived using the BS approach were consistently higher than those using the CTP approach. The association for CHE was found to be weak by both methods. The pattern of incidence and intensity of impoverishment was also similar to that of CHE.

**Table 3:** Incidence and intensity of catastrophic health spending, impoverishment and concentration index in India, 2004–18

Variables	Health Services			Hospitalisation			Outpatient		
	2004	2014	2018	2004	2014	2018	2004	2014	2018
OOP payment of household in 30 days at current prices (95% CI) in INR	746 (729–763)	2012 (1973–2051)	1995 (1961–2029)	487 (472–502)	1697 (1659–1734)	1609 (1582–1636)	615 (596–633)	1634 (1587–1681)	1765 (1711–1820)
OOP payment of household in 30 days at 2018 prices (95% CI) in INR	1910 (1867–1953)	2381 (2334–2427)	1995 (1961–2029)	1258 (1219–1298)	2019 (1975–2063)	1609 (1582–1636)	1566 (1520–1613)	1927 (1873–1982)	1765 (1711–1820)
Incidence of CHE based on capacity- to-pay approach (among all households)	12.45	13.43	9.10	3.42	4.42	2.92	9.26	9.07	6.25
Intensity of CHE based on capacity- to-pay approach	1.25	1.71	1.31	1.05	1.61	1.09	1.22	1.65	1.33
Incidence of CHE based on budget share	20.59	24.38	17.44	6.13	9.22	6.32	16.04	17.26	12.08
Correlation coefficient of intensity of CHE from budget share and capacity-to-pay approach	0.69	0.56	0.6	0.60	0.54	0.57	0.64	0.58	0.59
Incidence of Impoverishment due to health spending (among all households)	4.83	5.09	3.32	1.16	1.37	0.98	3.49	3.43	2.23
Concentration index of CHE (capacity to pay-all households)	−0.16	−0.18	−0.22	−0.17	−0.16	−0.22	−0.15	−0.2	−0.22
Standard error of CHE (capacity to pay)	0.009	0.012	0.012	0.014	0.014	0.013	0.011	0.012	0.016
Concentration index of CHE (budget share-all households)	0.02	−0.01	−0.03	0.06	0.02	−0.02	0.02	−0.02	−0.03
Standard error of CHE (budget share)	0.007	0.008	0.008	0.008	0.008	0.008	0.039	0.011	0.011

CHE and impoverishment had a strong socio-economic gradient in all three periods **(**Table 4**)**. For example, in 2018, about 15% of households in the poorest MPCE quintile incurred CHE, compared to 6% in the richest MPCE quintile and the pattern was similar over time. However, during 2004–14, there was an increase in incidence of CHE and impoverishment among poorest and poorer MPCE quintile but declined across all other MPCE quintiles. During 2014–2018, the incidence of CHE and impoverishment in health services has declined across each MPCE quintile. In last 14 years (2004–18), the decline in incidence of CHE was slower in the poorest MPCE quintile than in all the others. The differences in CHE between the richest and poorest MPCE quintiles was 6.9% in 2004, 11.26% in 2014 and 8.16% in 2018. Furthermore, CHE and impoverishment both showed a strong variation with the socio-demographic characteristics of households over time. Both CHE and impoverishment were consistently higher in rural than urban areas and declined over time. Households with coverage of any insurance had lower CHE and impoverishment than those households without insurance coverage. CHE and impoverishment by type of employment showed a lower prevalence in waged and salaried households and a higher prevalence in labourer households. Similarly, households with at least one elderly member had a high prevalence of CHE and impoverishment over time. Households headed by persons without any education had higher CHE and impoverishment while it was lowest among those with higher secondary education or above, in all three period. Both CHE and impoverishment were higher in female-headed households. Religious variations in CHE were not large in 2004 and 2014 but widened by 2018. In 2018, CHE was lowest among Sikhs and highest among “other religious groups” followed by Hindus. On average, household incurring CHE have incurred 125% of their capacity to pay in 2004, 171% in 2014 and 131% in 2018 (intensity of CHE).

**Table 4:** Incidence and intensity of catastrophic health spending and impoverishment by socio-economic and demographic characteristics in India, 2004–18

	Incidence of Catastrophic Health Spending (%)			Intensity of Catastrophic Health Spending			Impoverishment (%)		
	2004	2014	2018	2004	2014	2018	2004	2014	2018
**MPCE Quintile**									
Poorest	17.95	21.94	14.51	2.50	3.40	1.84	8.83	10.44	6.53
Poorer	13.15	14.24	9.46	0.82	0.89	0.92	4.99	5.52	3.36
Middle/Secondary	11.90	11.72	8.47	0.76	0.62	0.76	4.15	3.59	2.74
Richer	11.63	11.36	6.88	0.74	0.60	0.59	3.65	3.04	1.80
Richest	11.05	10.68	6.35	0.58	0.55	0.62	3.17	2.71	1.73
**Place of residence**									
Rural	13.91	14.98	10.34	1.34	1.92	1.46	5.58	6.01	3.94
Urban	8.67	10.22	6.56	0.87	1.08	0.83	2.89	3.17	2.04
**Covered by any health insurance schemes**									
No Insurance coverage	12.49	13.45	9.00	1.25	1.86	1.40	4.86	5.19	3.35
Any insurance coverage	10.30	13.31	9.54	0.91	1.04	0.93	3.30	4.65	3.20
**Age of head of household**									
Lt 30	11.47	11.55	7.18	1.22	1.09	2.28	4.90	4.33	2.66
30–44	11.05	10.79	7.17	1.42	1.79	1.00	4.24	3.93	2.52
45–59	12.09	13.09	8.40	1.11	1.47	1.13	4.45	4.94	3.14
60+	16.84	20.06	16.50	1.21	2.17	1.44	6.63	7.95	6.00
**Sex of the head of household**									
Male	12.34	13.19	8.87	1.20	1.48	1.18	4.73	4.91	3.19
Female	13.35	15.20	10.76	1.60	3.20	2.08	5.67	6.39	4.27
**Educational Attainment of head of household**									
No education	12.96	15.06	10.37	1.51	2.44	1.52	5.51	6.33	3.97
up to Primary	12.77	14.46	9.53	1.21	1.43	1.06	4.84	5.32	3.46
Middle/Secondary	12.95	13.20	8.75	1.03	1.24	1.50	4.58	4.62	3.12
Higher secondary	9.03	9.24	7.36	0.74	1.15	0.95	3.03	3.14	2.53
**Type of employment of household**									
Labour	13.53	14.45	9.24	1.65	2.05	1.83	5.72	6.08	3.52
Self Employed	7.12	10.32	7.22	0.79	1.42	0.76	2.24	3.26	1.91
Wage/salary	12.52	13.33	9.28	1.01	1.38	1.12	4.56	4.85	3.56
Others	14.37	19.12	12.01	1.29	2.75	1.64	5.96	8.17	4.59
**Any elderly member in the household**									
No	10.95	11.24	7.32	1.15	1.41	1.12	4.26	4.21	2.65
Yes	16.64	19.40	15.19	1.42	2.19	1.62	6.42	7.47	5.61
**Religion of household**									
Hindu	12.09	13.15	8.94	1.25	1.76	1.33	4.70	5.01	3.28
Muslim	14.90	14.91	9.83	1.23	1.52	1.08	6.06	5.71	3.73
Christian	14.72	14.26	9.55	1.50	1.14	0.92	4.86	5.54	3.13
Sikh	13.28	16.04	7.40	0.86	0.62	0.52	4.08	4.58	1.92
Others	9.32	11.43	15.50	1.36	4.22	3.57	3.71	3.21	4.45
**Total**	12.45	13.43	9.10	1.25	1.71	1.31	4.83	5.09	3.32

**Fig. 2:**
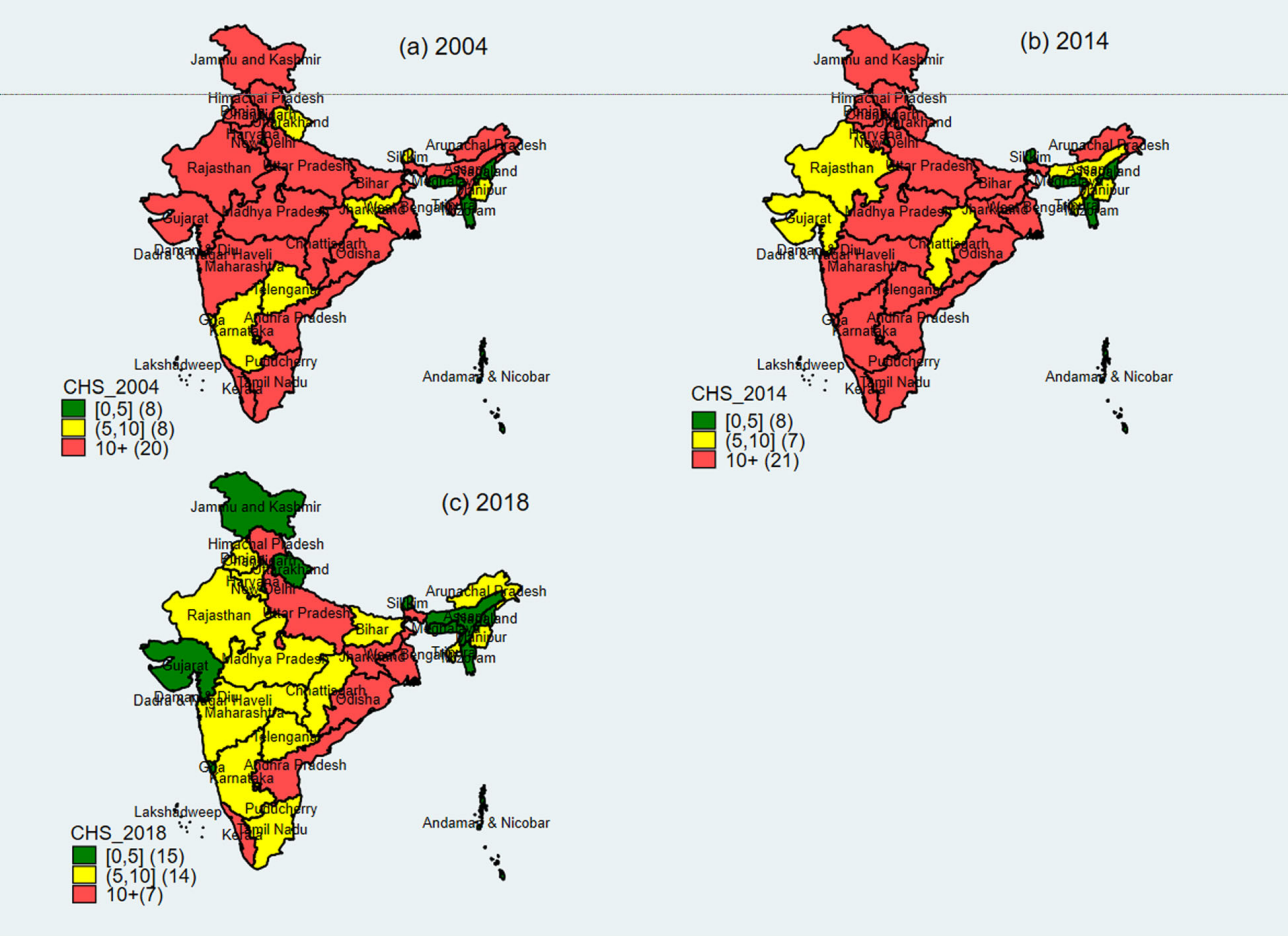
State pattern of catastrophic health spending (incidence) based on Capacity to Pay, 2004–18. Source: Author’s prepared map using their estimates

Table 5 presents the state pattern in incidence of CHE and impoverishment in India. In all three time period, the state variations in CHE and impoverishment were large. In general most of the states has shown increase in incidence of CHE and impoverishment during 2004–14 and declining pattern during 2014–18. The incidence of CHE was over 10% in 19 states/union territories in India in 2004 and it was in 21 states/union territories in 2014. By 2018, 11 of the states had incidence of CHE over 10%; it was higher in the poorer and developed states. In 2004, the incidence of CHE was highest in Kerala (22%) while in 2014 it was highest in Odisha (22%). In 2018, incidence of CHE was highest in Odisha, followed by Kerala, and lowest in Dadra and Nagar Haveli. Similarly, in 2018, impoverishment was highest in Odisha. In general, the extent of impoverishment was higher in the poorer states. Many of the states experienced reduction in impoverishment and incidence of CHE over time **(**Fig. 2**)**.

**Table 5:** Incidence of catastrophic health expenditure and impoverishment in states of India, 2004–18

		Incidence of CHE (%)		Impoverishment (%)		
States	2004	2014	2018	2004	2014	2018
Dadra & Nagar Haveli	4.02	7.20	0.49	1.46	0.94	0.09
Daman & Diu	2.33	2.33	0.49	1.09	0.20	0.13
Nagaland	4.75	1.39	0.99	1.15	0.09	0.13
Meghalaya	1.19	2.38	1.06	0.52	0.08	0.14
Pondicherry	7.06	10.56	1.74	1.62	2.81	0.84
Andaman & Nicobar	2.31	4.14	2.22	1.13	1.62	0.86
Sikkim	5.92	2.38	2.60	2.30	0.61	0.23
Mizoram	1.16	2.13	2.73	0.25	0.25	0.72
Goa	6.97	13.73	2.90	2.70	2.89	0.47
Uttarakhand	8.83	12.51	3.41	3.76	5.31	0.61
Assam	10.96	9.05	3.55	3.75	3.27	1.81
Delhi	1.04	2.40	3.80	0.35	1.10	0.65
Gujarat	10.47	5.97	3.97	3.26	1.94	1.27
Jammu & Kashmir	12.87	12.17	4.76	3.37	4.09	1.19
Lakshadweep	10.94	10.01	4.77	3.27	2.24	1.55
Telangana	9.94	15.77	5.28	4.32	6.37	1.44
Manipur	5.09	9.76	5.40	1.42	3.98	1.80
Chandigarh	3.55	3.36	5.49	1.04	0.18	0.74
Bihar	11.13	13.93	5.78	5.29	7.06	1.85
Karnataka	8.72	13.08	5.95	3.47	5.35	1.55
Haryana	14.06	10.22	5.97	5.24	2.20	1.76
Tamil Nadu	10.89	10.97	6.12	3.95	3.37	2.24
Rajasthan	12.42	9.97	6.69	5.46	3.02	2.28
Punjab	13.30	15.60	7.08	3.75	4.73	1.79
Madhya Pradesh	10.90	13.76	7.82	4.42	6.03	3.20
Tripura	15.78	8.54	7.94	5.22	2.60	2.79
Chhattisgarh	13.07	8.75	8.13	5.89	3.47	3.22
Maharashtra	12.32	11.12	9.10	4.80	3.75	3.42
India	12.45	13.43	9.10	4.83	5.09	3.32
Arunachal Pradesh	12.59	19.59	9.16	5.06	7.85	4.59
Himachal Pradesh	16.30	12.44	10.27	6.60	5.60	3.41
Andhra Pradesh	12.63	15.90	12.31	4.75	4.66	3.96
West Bengal	14.14	18.45	12.50	5.36	6.92	4.26
Jharkhand	7.98	11.03	13.34	3.05	4.25	4.46
Uttar Pradesh	15.39	16.25	13.52	6.22	6.42	5.88
Kerala	22.32	16.37	16.55	7.75	4.49	5.50
Odisha	14.03	21.60	16.78	5.48	10.88	7.04

### Concentration index and concentration curves of CHE

**Fig. 3:**
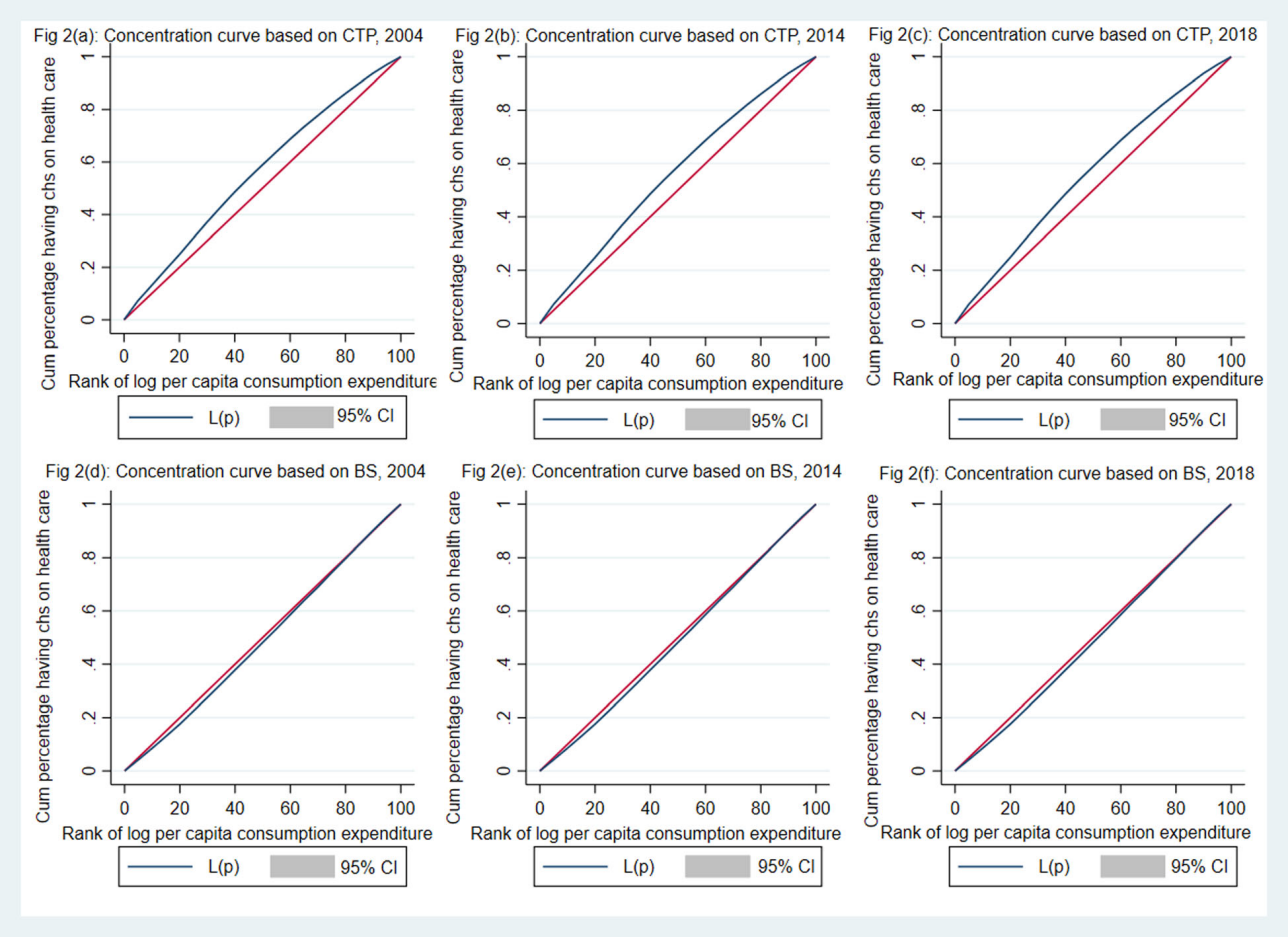
Concentration curves of catastrophic health spending on inpatient care using capacity-to-pay and budget share approach, 2004–18

Using the CTP approach, the concentration index of CHE for health services was − 0.16 in 2004, − 0.18 in 2014, and − 0.22 in 2018 (Table 6). The findings suggest that catastrophic health expenditure was concentrated among poorer households. However, when using the BS approach, the concentration index was close to zero for each of the services over all three periods (Table 2). The concentration curve (CC) of CHE for hospitalisation services (using the CTP approach) shifted upward over time, suggesting that the concentration of CHE increased among poorer households. When using the BS approach, it almost coincides with the diagonal line, suggesting that CHE is distributed equally among the population **(**Fig. 3**)**. Thus, the CC, when using the BS approach, does not capture the equity consideration of CHE. The pattern was similar for hospitalisation and outpatient services. The state pattern of concentration index over three point of time is robust. In most of the states, the concentration index is negative suggesting that the catastrophic health spending is concentrated among poor. The state variation in concentration index of CHE is large at all three point of time. Mizoram continued to have high CI in all three period. The CI was lower in Goa and Tamil Nadu.

**Table 6:** Concentration index of catastrophic health spending in states of India, 2004–18

Sates	2004		2014		2018	
	CI	SE	CI	SE	CI	SE
Andaman & Nicobar	−0.041	0.235	0.228	0.232	0.18	0.192
Andhra Pradesh	− 0.154***	0.028	− 0.137***	0.042	− 0.136***	0.041
Arunachal Pradesh	−0.222**	0.073	−0.107	0.092	− 0.225***	0.05
Assam	−0.138***	0.038	−0.342***	0.05	− 0.187**	0.067
Bihar	− 0.103***	0.032	− 0.189***	0.047	− 0.187***	0.05
Chandigarh	− 0.566**	0.179	NC	NC	−0.468	0.294
Chhattisgarh	−0.190***	0.035	−0.045	0.107	−0.113*	0.049
Dadra & Nagar Ha	−0.397	0.335	−0.376	0.18	NC	NC
Daman & Diu	−0.441*	0.177	−0.398	0.373	−0.294	0.298
Delhi	−0.014	0.138	−0.128	0.133	−0.223	0.125
Goa	−0.396*	0.187	0.286	0.223	−0.005	0.122
Gujarat	−0.228***	0.04	−0.111	0.057	−0.091	0.065
Haryana	−0.110*	0.05	−0.086	0.075	−0.240**	0.079
Himachal Pradesh	−0.168***	0.031	−0.190***	0.052	−0.229***	0.057
**India**	−0.160***	**0.007**	−0.177***	**0.01**	−0.217***	**0.01**
Jammu & Kashmir	−0.292***	0.044	−0.206**	0.066	−0.370***	0.105
Jharkhand	−0.091	0.055	−0.139	0.095	−0.136***	0.041
Karnataka	−0.159***	0.036	−0.167***	0.041	−0.263***	0.052
Kerala	−0.257***	0.02	−0.06	0.044	−0.129***	0.028
Lakshadweep	−0.085	0.122	−0.075	0.223	0.042	0.131
Madhya Pradesh	−0.111***	0.034	−0.186***	0.035	−0.227***	0.043
Maharashtra	−0.194***	0.028	−0.163***	0.035	−0.331***	0.034
Manipur	0.072	0.071	−0.326***	0.057	−0.295***	0.075
Meghalaya	0.067	0.142	−0.31	0.182	−0.569*	0.274
Mizoram	−0.465*	0.232	−0.314	0.219	−0.555***	0.161
Nagaland	−0.432	0.268	−0.565	0.412	−0.025	0.185
Orissa	−0.120***	0.027	−0.167***	0.028	−0.221***	0.025
Pondicherry	0.14	0.12	−0.208	0.15	−0.071	0.108
Punjab	−0.181***	0.038	−0.222***	0.058	−0.266***	0.059
Rajasthan	−0.121***	0.029	−0.119*	0.048	−0.146**	0.048
Sikkim	−0.176	0.095	0.029	0.123	−0.108	0.146
Tamil Nadu	−0.183***	0.03	−0.07	0.037	−0.201***	0.039
Telangana	−0.225***	0.037	−0.156**	0.058	−0.196***	0.058
Tripura	−0.114*	0.048	−0.203*	0.09	−0.343***	0.07
Uttar Pradesh	−0.108***	0.019	−0.131***	0.022	−0.123***	0.025
Uttarakhand	0.02	0.084	−0.173	0.1	−0.134	0.08
West Bengal	−0.186***	0.024	−0.157***	0.028	−0.181***	0.031

Note: *p < 0.05, **p < 0.01, ***p < 0.001. *NC*: *Not computed due to lower sample size*

### Determinants of CHE and impoverishment in India

Two regression models were estimated in order to understand the significant predictors of CHE and impoverishment **(**Table 7**)**. Estimates were derived from the polled data from the three periods: 2004, 2014, and 2018. Model 1 presents the odds ratio (OR) and 95% confidence interval of CHE, and model 2 presents the OR and the 95% confidence interval of impoverishment. The probability of incurring CHE was lower in urban than in rural areas [OR: 0.37, 95% CI: 0.35, 0.39]. The probability of incurring CHE decline with MPCE quintile; 66% lower among the richer MPCE quintile [OR: 0.34, 0.32–0.37] and 78% lower among the richest MPCE quintile [OR: 0.22, 95% CI: 0.21, 0.24] than among the poorest households. The interaction between time and MPCE quintile was found to be lower in 2018 than in 2014, suggesting that CHE declined across economic class in the later period. However, the gap among MPCE quintiles widened over various time periods. Similarly, households with at least one elderly member were significantly more likely to incur CHE than households without an elderly member, and the intensity of CHE significantly increased between 2004 and 2018. Demographic characteristics (age and sex) of household heads was significantly associated with CHE. Female-headed households were more likely to incur CHE than male-headed households [OR: 1.14, 95% CI: 1.06–1.23]. Religious differentials in CHE were not significant, but Christians and the “other religions” group were significantly less likely to incur CHE than Hindus. The OR of incurring CHE was 2.80 for those using both inpatient and outpatient care, compared to those who only used inpatient care [95% CI: 2.66–2.95], and the gap has increased over time. The interaction between insurance coverage, MPCE quintile, and time suggests that insurance helped to reduce CHE among the richer and richest MPCE quintiles, while it remained similar among the poorer and middle MPCE quintiles.

**Table 7:** Result of logistic regression analyses showing significant predictors of catastrophic health spending and impoverishment in India

Variables	CHE			IMP		
	OR	95% CI	***p***-value	OR	95% CI	p-value
**Place of residence**						
Rural (R)	1			1		
Urban	0.37	0.35–0.39	0.00	0.31	0.29–0.34	0.00
**MPCE Quintile**						
Poorest (R)	1			1		
Poorer	0.54	0.50–0.58	0.00	0.50	0.46–0.55	0.00
Middle	0.40	0.37–0.43	0.00	0.37	0.33–0.4	0.00
Richer	0.34	0.32–0.37	0.00	0.28	0.25–0.31	0.00
Richest	0.22	0.21–0.24	0.00	0.16	0.15–0.18	0.00
**Household Size**						
1–4 (R)	1			1		
5–7	0.61	0.58–0.65	0.00	0.53	0.50–0.57	0.00
8+	0.40	0.38–0.43	0.00	0.33	0.30–0.36	0.00
**Any elderly member in household**						
No elderly member in the household (R)						
Any elderly member in the household	1.20	1.12–1.28	0.00	1.14	1.04–1.25	0.01
**Main employment of household**						
Labourer Household (R)	1			1		
Wage/salary	0.94	0.86–1.04	0.22	1.04	0.91–1.2	0.55
Self Employed	1.11	1.05–1.17	0.00	1.08	1.01–1.16	0.04
Others	1.35	1.24–1.46	0.00	1.50	1.35–1.67	0.00
**Age of head of household**						
Lt 30 (R)						
30–44	1.22	1.14–1.32	0.00	1.22	1.10–1.35	0.00
45–59	1.44	1.34–1.55	0.00	1.44	1.30–1.59	0.00
60+	1.56	1.42–1.72	0.00	1.60	1.41–1.83	0.00
**Sex of the head of household**						
Male (R)	1			1		
Female	1.14	1.06–1.23	0.00	1.20	0.15–1.33	0.00
**Educational level of household**						
No education of head of household (R)	1			1		
Up to Primary	1.03	0.98–1.09	0.26	1.03	0.95–1.11	0.47
Middle/Secondary	1.19	1.12–1.27	0.00	1.16	1.07–1.26	0.00
Higher secondary	1.28	1.17–1.39	0.00	1.20	1.06–1.34	0.00
**Religion**						
Hindus (R)	1			1		
Muslim	1.05	0.98–1.12	0.16	1.04	0.95–1.14	0.41
Christian	0.81	0.72–0.92	0.00	0.99	0.83–1.19	0.95
Sikh	1.11	0.94–1.33	0.23	1.20	0.93–1.54	0.17
Ohers	1.00	0.85–1.18	0.99	0.91	0.72–1.16	0.46
**Inpatient and outpatient care**						
Availed inpatient care only	1		1	1		
Availed outpatient care only	1.33	1.25–1.4	0.00	1.28	1.18–1.39	0.00
Availed both inpatient and outpatient care	2.80	2.66–2.95	0.00	3.12	2.91–3.34	0.00
**Insurance coverage**						
No (R)	1			1		
Yes	0.89	0.76–1.03	0.11	0.93	0.74–1.17	0.55
**Time**						
2004 (R)	1			1		
2014	0.80	0.71–0.91	0.00	0.67	0.57–0.80	0.00
2018	0.56	0.50–0.63	0.00	0.41	0.63–0.49	0.00
**Interaction between Time and MPCE Quintile**						
2014*Poorer	0.89	0.81–0.99	0.03	0.90	0.79–1.02	0.10
2018*Poorer	0.83	0.76–0.92	0.00	0.82	0.73–0.93	0.00
2014*Middle	0.90	0.81–0.99	0.03	0.82	0.72–0.94	0.00
2018*Middle	0.82	0.74–0.9	0.00	0.75	0.66–0.85	0.00
2014*Richer	0.80	0.72–0.88	0.00	0.71	0.62–0.81	0.00
2018*Richer	0.66	0.60–0.73	0.00	0.72	0.63–0.82	0.00
2014*Richest	1.01	0.90–1.13	0.87	0.91	0.78–1.06	0.21
2018*Richest	0.71	0.65–0.79	0.00	0.76	0.66–0.88	0.00
**Interaction of Time, Insurance and MPCE Quintile**						
2014* Insurance*Poorer	1.10	0.93–1.32	0.27	1.04	0.83–1.31	0.72
2018* Insurance*Poorer	1.03	0.89–1.2	0.66	0.98	0.80–1.2	0.82
2014* Insurance*Middle	1.06	0.89–1.26	0.52	1.06	0.84–1.34	0.62
2018* Insurance*Middle	0.94	0.82–1.09	0.43	0.95	0.78–1.16	0.61
2014* Insurance*Richer	1.05	0.89–1.25	0.54	1.21	0.97–1.51	0.09
2018* Insurance*Richer	1.06	0.92–1.22	0.45	0.91	0.74–1.11	0.34
2014* Insurance*Richest	0.79	0.67–0.92	0.00	0.72	0.58–0.9	0.01
2018* Insurance*Richest	0.84	0.74–0.96	0.01	0.74	0.62–0.9	0.00
Puuedo R^2^	0.14			0.15		

The pattern of impoverishment was similar to CHE (model 2). Rural households, poorest and poorer households, and female-headed households were significantly more likely to incur impoverishment due to health expenditure in India. The pattern of the different characteristics for the study sample was similar to CHE. However, the interaction between time and MPCE quintile was significant, and shows that the gap widened between the poorest and richest categories between 2004 and 2018.

## Discussion and conclusion

Reliable estimates of catastrophic health expenditure and impoverishment are paramount for monitoring global, national and state developmental goals. While estimates for India have been provided time and again, they differ to a large extent owing to data and methodological limitations. In this context, this paper has addressed the data and methodological limitations in estimating CHE in India and makes both methodological and empirical contributions. Methodologically, it has demonstrated how to estimate catastrophic health expenditure and impoverishment with limited information about consumption expenditure by using the capacity-to-pay approach. Empirically, this is the first-ever attempt to provide comprehensive estimates on incidence and intensity of catastrophic health expenditure and impoverishment in India over the last 14 years. The followings are the salient findings of the study.

First, our results demonstrate that it is possible to derive robust estimates of CHE and impoverishment using the CTP approach, even with the limited data on consumption expenditure available from health surveys in India. Second, our findings suggest CHE and impoverishment in India has increased during 2004–14 and declined between 2014 and 2018. Third, the estimates of CHE derived using the proposed method and capacity-to-pay approach have a strong economic gradient. Literature suggests that OOP and CHE are regressive and affect the poor the most, and that reliable measures need to be sensitive to the lower strata of the population. Our results support the findings that CHE and impoverishment among the poorest and poorer households remained consistently high over three point of time. Fourth, although there has been a decline in OOP payment, increase in reimbursement, declining in CHE and impoverishment, the concentration index was − 0.16 in 2004 and increased to − 0.22 by 2018 suggesting that the CHE is concentrated among poor and has increased over time. Fifth, when comparing the estimates of CHE using CTP and BS approaches, we found that CTP is robust as it captures equity concerns. The BS method tends to overestimate CHE compared to CTP approach. The extent of CHE based on the BS method was higher among richer people than among the poorer and the concentration index was close to zero. These estimates are questionable as the poor bear the burden of financial catastrophe. The CHE derived using the capacity-to-pay approach was twice as high among the poorest population as among the richest population. Impoverishment due to health expenditure was equally high. The association of BS and CTP approaches is weak. Finally, our findings suggest that rural households, poor households, female-headed households, households with either outpatient care or both inpatient and outpatient care and households with at least one elderly member are more likely to incur CHE and face impoverishment due to health expenditure.

We have provided up-to-date estimates of CHE and impoverishment at household level with analyses from health surveys using the CTP approach at three point of time. To our knowledge, we have not come across similar studies in India. We made two improvements when deriving the estimates of CHE and impoverishment using the CTP approach. First, we derived the per capita food expenditure from the CSs and used the HSs to derive the subsistence expenditure. Our assumption of stability in the share of food expenditure in the consumption basket in the short run is rational. The distribution of food expenditure can be taken from independent consumption surveys and then integrated into health surveys that lack such information. The consumption and health survey at two point of time are close to each other while that of 2017–18 health survey was 6 with a gap of 6 years. Since no latest consumption survey was available between we kept the distribution constant and it may marginally have higher CHE for 2018. Second, we adjusted the household size separately for rural and urban areas. Furthermore, we assumed that households whose total consumption expenditure was lower than the subsistence expenditure and incurring OOP expenditure on health services were incurring CHE. This exercise can be replicated elsewhere (any other country) with similar information. We provide robust estimates of CHE across MPCE quintile using the CTP approach and findings are in expected direction. The weak correlation in estimates of CHE using the CTP and BS approaches is implicit in the method used. The fact that poor people meet their health expenditure largely by borrowing and selling assets (a form of distress financing) and reducing spending on essential items, while rich people financed health expenditure from their income or past savings or through insurance, is reflected in the high CHE among poorest and poorer households and in the high concentration index is captured in CTP estimates.

Two of the paper appeared in Bulletin of WHO amply discussed the limitations of budget share method in estimating CHE and inconsistency in estimates due to use of varying method and survey [2, 4]. The use of budget share method underestimate CHE among the poor. Earlier estimates based on budget share approach in India suggest higher CHE among richer MPCE quintile than poorer quintile and pro-rich distribution of CHE [30, 33]. Our findings is consistent with high CHE among poor. Further, previous studies have invariably cited lack of data on food expenditure for estimating CHE using health surveys (NSS) [36]. We have overcome this limitation and provided robust estimates for India. Our estimates using the above method and capacity to pay approach provides consistent estimates across MPCE quintile and captures the equity concern.

We have put forward some plausible explanation for declining CHE and impoverishment in India. Though the mean medical expenditure can increase with greater income, it may not necessarily involve OOP payments if insurance coverage or public spending on health also increases. Evidence suggests that in the last decade, there has been an increase in insurance coverage in India and the mean reimbursement at constant prices has increased over time. Our findings support that health insurance has reduced catastrophic health expenditure and impoverishment among richer and richest households, but not among the poorest, poorer, and middle MPCE quintiles. Essential drugs, as well as the prices of certain medical services such as stents, have been regulated may have reduced CHE.

The study has the following limitations. First, it did not take into account the impact of the *Ayushman Bharat Yojana (ABY)* launched in 2018. The ABY is the largest-ever nationwide health scheme implemented to protect the poor and the needy from financial catastrophe. It aims to provide health protection to over 100 million families, accounting for over 40% of the population (www.ayushmanbharat.co.in). The effects of ABY may well be captured in the next round of survey. These estimates of CHE and impoverishment may serve as baseline estimates for ABY. Second, though CHE provides numerical estimates of the financial hardship faced by households, it has not captured the extent of distress financing such as borrowing and selling assets. Third, our estimates of 2018 is based on the distribution of 2011–12 which may overestimate the CHE and impoverishment marginally.

Based on our results, we suggest that studies on CHE should be based on uniform approach (the CTP method). The NSS health survey (schedule 25.0) should include a smaller module of consumption expenditure. Increasing investments in public health can reduce catastrophic health expenditure and impoverishment.

## Supplementary Information

**Additional file 1.** Subsistence expenditure as share of household consumption expenditure, 2004–18.

## Data Availability

The dataset used and analysed for the current study is publicly available.

## Abbreviations

CHE: Catastrophic Health Expenditure
CTP: Capacity-to-pay
BS: Budget share
OOP: Out-of-Pocket Payment
HSs: Health surveys
CSs: Consumption survey
NSS: National Sample Survey
NHM: National Health Mission
ABY: Ayushman Bharat Yojana
SE: Subsistence expenditure
CC: Concentration curve
CI: Concentration index
