# Supplementary material for: Addressing data and methodological limitations in estimating catastrophic health spending and impoverishment in India, 2004–18
Source: Int J Equity Health. 2021 Mar 20;20:85. doi: 10.1186/s12939-021-01421-6 (PMC7981828; doi:10.1186/s12939-021-01421-6)
Supplement: Supplementary file 1 — Additional file 1. Subsistence expenditure as share of household consumption expenditure, 2004–18. [file 12939_2021_1421_MOESM1_ESM.docx]

**Appendix 1: Subsistence expenditure as share of household consumption expenditure, 2004-18.**

| **State / India** | **Rural** | | | **Urban** | | |
| --- | --- | --- | --- | --- | --- | --- |
|  | **2004** | **2014** | **2018** | **2004** | **2014** | **2018** |
| Andaman & Nicobar | 48.47 | 19.44 | 16.93 | 33.18 | 15.13 | 12.61 |
| Andhra Pradesh | 47.49 | 33.06 | 34.85 | 32.67 | 20.79 | 18.08 |
| Arunachal Pradesh | 38.08 | 38.80 | 37.60 | 32.70 | 19.63 | 17.24 |
| Assam | 49.07 | 42.92 | 44.31 | 33.24 | 20.52 | 21.97 |
| Bihar | 45.04 | 44.86 | 44.87 | 30.08 | 28.44 | 30.60 |
| Chandigarh | 43.15 | 27.40 | 24.75 | 34.84 | 21.93 | 16.44 |
| Chhattisgarh | 47.31 | 53.05 | 48.19 | 26.92 | 26.44 | 26.73 |
| Dadra & Nagar Haveli | 41.57 | 45.73 | 46.80 | 19.95 | 24.88 | 22.22 |
| Daman & Diu | 32.71 | 22.57 | 36.98 | 28.77 | 24.76 | 19.48 |
| Delhi | 38.04 | 16.68 | 21.68 | 28.90 | 16.26 | 14.46 |
| Goa | 50.50 | 17.99 | 20.83 | 28.35 | 13.12 | 14.19 |
| Gujarat | 49.18 | 31.97 | 36.09 | 28.50 | 15.62 | 17.31 |
| Haryana | 39.54 | 23.38 | 24.43 | 30.46 | 13.74 | 12.43 |
| Himachal Pradesh | 36.64 | 29.44 | 28.19 | 29.55 | 17.76 | 16.31 |
| **India** | **44.35** | **39.88** | **39.73** | **28.28** | **18.31** | **18.96** |
| Jammu & Kashmir | 43.39 | 30.69 | 31.58 | 30.16 | 18.47 | 17.37 |
| Jharkhand | 48.85 | 52.61 | 50.93 | 26.40 | 19.04 | 19.78 |
| Karnataka | 40.66 | 35.65 | 34.77 | 28.14 | 16.11 | 19.71 |
| Kerala | 34.88 | 23.86 | 24.74 | 28.28 | 16.58 | 18.82 |
| Lakshadweep | 31.78 | 18.31 | 17.17 | 33.39 | 12.48 | 17.55 |
| Madhya Pradesh | 40.35 | 47.48 | 48.96 | 27.33 | 23.68 | 20.47 |
| Maharashtra | 46.45 | 30.12 | 35.00 | 27.02 | 14.95 | 15.20 |
| Manipur | 41.13 | 34.99 | 33.35 | 30.66 | 31.37 | 27.36 |
| Meghalaya | 47.31 | 35.31 | 34.09 | 29.40 | 17.61 | 16.78 |
| Mizoram | 48.64 | 31.94 | 30.57 | 30.57 | 14.69 | 15.85 |
| Nagaland | 41.05 | 26.09 | 28.12 | 29.45 | 17.46 | 19.07 |
| Orissa | 44.42 | 50.87 | 54.13 | 36.74 | 33.99 | 28.34 |
| Pondicherry | 35.62 | 19.37 | 22.28 | 26.94 | 15.43 | 15.37 |
| Punjab | 38.00 | 27.96 | 21.80 | 29.86 | 16.95 | 16.03 |
| Rajasthan | 39.65 | 36.75 | 32.73 | 31.62 | 15.50 | 18.34 |
| Sikkim | 43.15 | 32.40 | 36.53 | 32.37 | 15.21 | 20.27 |
| Tamil Nadu | 49.16 | 37.01 | 38.02 | 30.94 | 18.12 | 20.68 |
| Telangana | 45.17 | 33.66 | 39.09 | 27.09 | 17.09 | 23.58 |
| Tripura | 51.25 | 40.80 | 41.68 | 41.29 | 25.46 | 24.62 |
| Uttar Pradesh | 44.23 | 46.13 | 45.61 | 29.55 | 24.30 | 24.42 |
| Uttarakhand | 45.12 | 31.96 | 30.83 | 33.79 | 21.44 | 21.93 |
| West Bengal | 43.00 | 43.53 | 45.64 | 34.64 | 19.37 | 23.23 |
